# Supplementary material for: A Systematic Review of Endoscopic Treatments for Concomitant Malignant Biliary Obstruction and Malignant Gastric Outlet Obstruction and the Outstanding Role of Endoscopic Ultrasound-Guided Therapies
Source: Cancers (Basel). 2023 Apr 30;15(9):2585. doi: 10.3390/cancers15092585 (PMC10177271; doi:10.3390/cancers15092585)
Supplement: Supplementary file 1 [file cancers-15-02585-s001.zip › cancers-2346484-supplementary.pdf]

Supplementary Materials

Methods

PICO assessment:

> **P**: patients with concomitant malignant extrahepatic biliary obstruction and gastric outlet obstruction (GOO)
>
> **I**: concomitant endoscopic biliary drainage (EUS-guided) and endoscopic treatment of MGOO
>
> **C**: *no comparison*
>
> **O**: technical success, clinical success and safety (rate of adverse events).

Search question:

> What are efficacy and safety in patients with concomitant malignant extrahepatic biliary obstruction and gastric outlet obstruction undergoing EUS-guided biliary drainage and duodenal stenting/EUS-guided gastroenteroanastomosis?

Search strategy

Followed string were used:

('cholestasis'/exp OR (cholestas* OR ((bil* OR choledoc*) NEAR/3 (obstruction* OR occlusion* OR stenos* OR strictur* OR stasi* OR obliteration*))):ab,ti) AND ('endoscopic ultrasonography'/exp OR 'endoscopic ultrasonography guided hepaticogastrostomy'/exp OR 'endoscopic ultrasonography guided biliary drainage'/exp OR (EUS* OR echoendoscope* OR endosonograph* OR ((echograph* OR endoscopic*) NEAR/3 (endoscopic* OR ultrasound* OR echograph*))):ab:ti) AND ('pylorus stenosis'/exp OR (pylorostenos* OR ((pylor* OR gastric* OR stomach* OR duodenal*) NEAR/3 (obstruction* OR stenos* OR stricture*))):ab,ti OR 'duodenum stenosis'/exp OR (duodenal-stenos*):ab,ti OR 'duodenal stricture'/exp) AND ('malignant neoplasm'/exp OR (cancer* OR ((malignant* OR neoplas* OR oncolog* OR tumor*) NEAR/3 (neoplas* OR tumor* OR maligna*))):ab,ti)

TABLES

Table S1. Technical features of MGOO treatment (duodenal stenting and EUS-GEA) among included studies

Study, year	Type of treatment	Type of stent	Technical success, %	Clinical success, %	AEs, n(%)
**Iwamuro, 2010[1]**	Duodenal stenting	Niti-S stent (Taewoong, Seoul, Korea, 20 mm in diameter and 80 mm in length)	100	100	0 (0)
**Maluf-Filho, 2012[2]**	Duodenal stenting	(WallFlex® 120/220 mm, Boston Scientific, Natick, MA, USA	100	60	0 (0)
**Rebello, 2012[3]**	Duodenal stenting	NA	100	100	0 (0)
**Tonozuka, 2013[4]**	Duodenal stenting	Niti-S ComViT,<br>TaeWoong Medical<br><br>Or<br>WallFlexTM duodenal stent (22 mm in diameter: 60–90 mm in length; Boston Scientific Japan, Tokyo, Japan)	100	100	0 (0)
**Ogura, 2016[5]**	Duodenal stenting	U.SEMS: Niti-S Duodenal Uncovered Metallic Stent–TaeWoong Medical, Seoul, Korea; Century Medical Inc., Tokyo, Japan	100	NA	0
**Sato, 2016[6]**	Duodenal stenting	Uncovered Niti- STM Pyloric Duodenal D-type stents<br>or<br>WallFlexTM Duodenal stents	100	NA	NA
**Hamada, 2018[7]**	Duodenal stenting	U-SEMS<br>FC-SEMS<br>PC-SEMS	100	NA	NA
**Debourdeau, 2021[8]**	Duodenal stenting	U-SEMS	NA	NA	NA
**Mangiavillano, 2021[9]**	Duodenal stenting	U-SEMS (Wallflex; Boston Scientific, Natick, Massachusetts, United States)<br>or<br>U-SEMS (Niti-S (Taewoong; Korea).	100	NA	0
**Canakis, 2022[10]**	EUS-GEA	LAMS, Axios-EC, (Boston Scientific)	95.6	100	0
**Sasaki, 2022[11]**	Duodenal stenting	Niti-S duodenal stent (Taewoong Medical)<br>or<br>WallFlex Duodenal Stent (Boston Scientific Japan),<br>or<br>Evolution Duodenal Controlled-Release Stent— uncovered (Cook Medical Japan)<br>or<br>NEXENT duodenal stent (Create Medic)<br>or<br>HANAROSTENT Naturfit Duo (Boston Scientific Japan)	NA	NA	NA

EUS-GEA=Endoscopic Ultrasound Gastroentero-anastomosis; PC-SEMS= Partially covered self-expandable metal stent; U-SEMS= uncovered self-expandable metal stent; MGOO= Malignant Gastric Outlet Obstruction; AEs= adverse events; C-SEMS= covered self-expandable metal stent; FCSEMS= fully covered self-expandable metal stent; NA= Not Available.

Table S2. Technical features of EUS-BD among studies

Study, year	Type of EUS-BD	Type of stent	Technical success, %	Clinical success, %	Adverse events, n (%)	Type of AEs	Definition of clinical success	Definition of technical success
**Iwamuro, 2010 [1]**	Transduodenal (EUS-CDS)	Polyethylene stent (7-Fr 50-mm-long Flexima stent; Boston Scientific, or 7-Fr 40-mm-long Zimmon stent; Wilson Cook)	100	100	1 (50)	Severe abdominal pain and fever	Resolution of obstructive jaundice or successful withdrawal of an external indwelling catheter for biliary decompression after EUS-guided biliary drainage	NA
**Maluf-Filho, 2012[2]**	Transgastric or transduodenal	WallFlex® 60/100 mm, Boston Scientific, Natick, MA, USA	100	60	3 (60)	Biliary fistula, duodeno-enteric fistula and cardiogenic shock (immediate post-procedure)	NA	complete stent placement
**Rebello, 2012[3]**	Transduodenal (EUS-CDS)	Wallfex® , Boston Scientific	100	85	0 (0)	-	NA	complete stent placement
**Tonozuka, 2013[4]**	Transduodenal or transgastric	WallFlex™ Boston Scientific;<br>Or<br>Niti-S biliary stent (20 mm in diameter: 100 mm in length; Taewoong Medical, Seoul, South Korea)<br>Or<br>HANAROSTENT® Biliary, M.I. Tech, Seoul, South Korea	100	100	2 (50)	Cholangitis	Elimination of jaundice and cholangitis, and whether oral ingestion could be resumed	NA
**Ogura, 2016[5]**	Transduodenal (n=13)<br>Transgastric (N=26)	EUS-CDS: metallic stent (10mm×6cm), Wallstent – Boston Scientific Japan, Tokyo, Japan; Bona stent – Sewoon Medical Co., Ltd., Seoul, Kore<br>EUS-HGS: metallic stent 10 mm × 10 cm, End-bare type, Niti-S Biliary Cover Stent – Tae-Woong Medical; Century Medical Inc., Tokyo, Japan)	NA	NA	8 (20.5)	NA	A decrease in bilirubin to < 75 % of pre-drainage levels within 30 days	Complete stent placement
**Sato, 2016[6]**	Transduodenal (n=16)<br>Or<br>Transgastric (n=1)	EUS-CDS: SEMS fully covered WallFlex™<br>Biliary RX Stents<br>EUS-HGS: fully covered NitiS™ biliary stents (Taewoong Medical, Seoul, Korea)	96.5	100	NA	NA	Improvements in serum liver enzyme levels and/or bilirubin levels in biliary drainage specimens and improvement in the symptoms and oral intake in gastroduodenal stentings	NA
**Hamada, 2018[7]**	EUS-CDS<br>EUS-HGS	SEMS	100	NA	7 (35)	Cholangitis 3<br>Cholangitis and Bleeding 1 Pancreatitis 1	>50% bilirubin decrease from the value before biliary drainage or normalization within two weeks	NA

						Bile leakage and Pneumoperitoneum 1<br>Migration 1		
**Debourdeau, 2021[8]**	EUS-HGS<br>EUS-CDS	PC-SEMS (Giobor Taewong)	100	NA	NA	NA	NA	NA
**Mangiavillano, 2021[9]**	EUS-GDS (n=14)<br>EUS-CDS (n=19)	LAMSs were Hot-Axios (Boston Scientific, Natick, Massachusetts, United States): 6mm× 8mm in 7 cases (30.4%), 8mm× 8mm in 1 case (4.4 %) and 10mm× 10mm in 15 cases (65.2%)	95.6	NA	0	-	As correct placement of the LAMS through the meshes of a duodenal SEMS.	A bilirubin level decrease > 15% after 24 hours from the LAMS placement.
**Canakis, 2022[10]**	EUS-HGS	FC-SEMS (VIABIL Biliary Endoprosthesis, W.L.<br>Gore & Associates, Flagstaff, Ariz)	100	72.7	3 (14.3)	Biliary stent Dislodgment (n=2)<br>Hepatic abscess (n=1)	Successful stent placement from the left intrahepatic duct and gastric wall to the gastrointestinal tract	A subsequent bilirubin decrease documented to be at least 50% without reintervention
**Sasaki, 2022[11]**	EUS-CDS<br>EUS-HGS	Duckbill-type metal stent (Kawasumi Duckbill Biliary Stent)<br>Or<br>Niti-SS type (Taewoong Medical)<br>Or<br>Spring Stopper (Taewoong Medical)<br>Or<br>EGIS biliary stent (SB-Kawasumi laboratories)	NA	NA	NA	NA	NA	NA

BD= biliary drainage; EUS-BD=Endoscopic Ultrasound biliary drainage; PC-SEMS= Partially covered self-expandable metal stent; MGOO= Malignant Gastric Outlet Obstruction; AEs= adverse events; C-SEMS= covered self-expandable metal stent; FCSEMS= fully covered self-expandable metal stent; NA= Not Available; LAMS = Lumen apposing metal stent; EUS-CDS= Endoscopic Ultrasound Choledocoduodenostomy; EUS-GDS= Endoscopic Ultrasound Gallbladderduodenostomy; EUS-HGS= Endoscopic Ultrasound Hepaticogastrostomy.

PRISMA Checklist

Section and Topic	Item #	Checklist item	Page number where item is reported
**TITLE**			
Title	1	Identify the report as a systematic review.	1

Section and Topic	Item #	Checklist item	Page number where item is reported
**ABSTRACT**			
Abstract	2	See the PRISMA 2020 for Abstracts checklist.	supplementary
**INTRODUCTION**			
Rationale	3	Describe the rationale for the review in the context of existing knowledge.	2
Objectives	4	Provide an explicit statement of the objective(s) or question(s) the review addresses.	2
**METHODS**			
Eligibility criteria	5	Specify the inclusion and exclusion criteria for the review and how studies were grouped for the syntheses.	5
Information sources	6	Specify all databases, registers, websites, organisations, reference lists and other sources searched or consulted to identify studies. Specify the date when each source was last searched or consulted.	5
Search strategy	7	Present the full search strategies for all databases, registers and websites, including any filters and limits used.	supplementary
Selection process	8	Specify the methods used to decide whether a study met the inclusion criteria of the review, including how many reviewers screened each record and each report retrieved, whether they worked independently, and if applicable, details of automation tools used in the process.	5
Data collection process	9	Specify the methods used to collect data from reports, including how many reviewers collected data from each report, whether they worked independently, any processes for obtaining or confirming data from study investigators, and if applicable, details of automation tools used in the process.	5
Data items	10a	List and define all outcomes for which data were sought. Specify whether all results that were compatible with each outcome domain in each study were sought (e.g. for all measures, time points, analyses), and if not, the methods used to decide which results to collect.	5-6
	10b	List and define all other variables for which data were sought (e.g. participant and intervention characteristics, funding sources). Describe any assumptions made about any missing or unclear information.	5-6
Study risk of bias assessment	11	Specify the methods used to assess risk of bias in the included studies, including details of the tool(s) used, how many reviewers assessed each study and whether they worked independently, and if applicable, details of automation tools used in the process.	5-6
Effect measures	12	Specify for each outcome the effect measure(s) (e.g. risk ratio, mean difference) used in the synthesis or presentation of results.	5-6
Synthesis methods	13a	Describe the processes used to decide which studies were eligible for each synthesis (e.g. tabulating the study intervention characteristics and comparing against the planned groups for each synthesis (item #5)).	5-6
	13b	Describe any methods required to prepare the data for presentation or synthesis, such as handling of missing summary statistics, or data conversions.	5-6
	13c	Describe any methods used to tabulate or visually display results of individual studies and syntheses.	5-6
	13d	Describe any methods used to synthesize results and provide a rationale for the choice(s). If meta-analysis was performed, describe the model(s), method(s) to identify the presence and extent of statistical heterogeneity, and software package(s) used.	5-6
	13e	Describe any methods used to explore possible causes of heterogeneity among study results (e.g. subgroup analysis, meta-regression).	5-6
	13f	Describe any sensitivity analyses conducted to assess robustness of the synthesized results.	5-6

Section and Topic	Item #	Checklist item	Page number where item is reported
Reporting bias assessment	14	Describe any methods used to assess risk of bias due to missing results in a synthesis (arising from reporting biases).	5-6
Certainty assessment	15	Describe any methods used to assess certainty (or confidence) in the body of evidence for an outcome.	5-6
**RESULTS**			
Study selection	16a	Describe the results of the search and selection process, from the number of records identified in the search to the number of studies included in the review, ideally using a flow diagram.	6–7 (Fig 1)
	16b	Cite studies that might appear to meet the inclusion criteria, but which were excluded, and explain why they were excluded.	6–7 (Fig 1)
Study characteristics	17	Cite each included study and present its characteristics.	7-8
Risk of bias in studies	18	Present assessments of risk of bias for each included study.	NA
Results of individual studies	19	For all outcomes, present, for each study: (a) summary statistics for each group (where appropriate) and (b) an effect estimate and its precision (e.g. confidence/credible interval), ideally using structured tables or plots.	8-9
Results of syntheses	20a	For each synthesis, briefly summarise the characteristics and risk of bias among contributing studies.	8-9
	20b	Present results of all statistical syntheses conducted. If meta-analysis was done, present for each the summary estimate and its precision (e.g. confidence/credible interval) and measures of statistical heterogeneity. If comparing groups, describe the direction of the effect.	8
	20c	Present results of all investigations of possible causes of heterogeneity among study results.	NA
	20d	Present results of all sensitivity analyses conducted to assess the robustness of the synthesized results.	NA
Reporting biases	21	Present assessments of risk of bias due to missing results (arising from reporting biases) for each synthesis assessed.	13
Certainty of evidence	22	Present assessments of certainty (or confidence) in the body of evidence for each outcome assessed.	8
**DISCUSSION**			
Discussion	23a	Provide a general interpretation of the results in the context of other evidence.	11-12-13
	23b	Discuss any limitations of the evidence included in the review.	13
	23c	Discuss any limitations of the review processes used.	13
	23d	Discuss implications of the results for practice, policy, and future research.	14
**OTHER INFORMATION**			
Registration and protocol	24a	Provide registration information for the review, including register name and registration number, or state that the review was not registered.	NA
	24b	Indicate where the review protocol can be accessed, or state that a protocol was not prepared.	NA

Section and Topic	Item #	Checklist item	Page number where item is reported
	24c	Describe and explain any amendments to information provided at registration or in the protocol.	NA
Support	25	Describe sources of financial or non-financial support for the review, and the role of the funders or sponsors in the review.	14
Competing interests	26	Declare any competing interests of review authors.	14
Availability of data, code and other materials	27	Report which of the following are publicly available and where they can be found: template data collection forms; data extracted from included studies; data used for all analyses; analytic code; any other materials used in the review.	14

PRISMA Abstract Checklist

Section and Topic	Item #	Checklist item	Reported (Yes/No)
**TITLE**			
Title	1	Identify the report as a systematic review.	Yes
**BACKGROUND**			
Objectives	2	Provide an explicit statement of the main objective(s) or question(s) the review addresses.	Yes
**METHODS**			
Eligibility criteria	3	Specify the inclusion and exclusion criteria for the review.	Yes
Information sources	4	Specify the information sources (e.g. databases, registers) used to identify studies and the date when each was last searched.	Yes
Risk of bias	5	Specify the methods used to assess risk of bias in the included studies.	No
Synthesis of results	6	Specify the methods used to present and synthesise results.	No
**RESULTS**			
Included studies	7	Give the total number of included studies and participants and summarise relevant characteristics of studies.	Yes
Synthesis of results	8	Present results for main outcomes, preferably indicating the number of included studies and participants for each. If meta-analysis was done, report the summary estimate and confidence/credible interval. If comparing	Yes

Section and Topic	Item #	Checklist item	Reported (Yes/No)
		groups, indicate the direction of the effect (i.e. which group is favoured).	
**DISCUSSION**			
Limitations of evidence	9	Provide a brief summary of the limitations of the evidence included in the review (e.g. study risk of bias, inconsistency and imprecision).	No
Interpretation	10	Provide a general interpretation of the results and important implications.	Yes
**OTHER**			
Funding	11	Specify the primary source of funding for the review.	No
Registration	12	Provide the register name and registration number.	No

REFERENCES

1. Iwamuro, M., H. Kawamoto, R. Harada, H. Kato, K. Hirao, O. Mizuno, E. Ishida, T. Ogawa, H. Okada, and K. Yamamoto, *Combined duodenal stent placement and endoscopic ultrasonography-guided biliary drainage for malignant duodenal obstruction with biliary stricture.* Dig Endosc, 2010. **22**(3): p. 236-40.
2. Maluf-Filho, F., F.A. Retes, C.Z. Neves, C.F.M. Sato, F.S. Kawaguti, R. Jureidini, U. Ribeiro Jr., and T. Bacchella, *Transduodenal endosonography-guided biliary drainage and duodenal stenting for palliation of malignant obstructive jaundice and duodenal obstruction.* J. Pancreas, 2012. **13**(2): p. 210-214.
3. Rebello, C., A. Bordini, A. Yoshida, B. Viana, P.E.N. Ramos, J.P. Otoch, L.M. Cirino, and E.L.A. Artifon, *A one-step procedure by using linear echoendoscope to perform EUS-guided choledochoduodenostomy and duodenal stenting in patients with irresectable periampullary cancer.* Endoscopic Ultrasound, 2012. **1**(3): p. 156-161.
4. Tonozuka, R., T. Itoi, A. Sofuni, F. Itokawa, and F. Moriyasu, *Endoscopic double stenting for the treatment of malignant biliary and duodenal obstruction due to pancreatic cancer.* Dig. Endosc., 2013. **25**: p. 100-108.
5. Ogura, T., Y. Chiba, D. Masuda, M. Kitano, T. Sano, O. Saori, K. Yamamoto, H. Imaoka, A. Imoto, T. Takeuchi, et al., *Comparison of the clinical impact of endoscopic ultrasound-guided choledochoduodenostomy and hepaticogastrostomy for bile duct obstruction with duodenal obstruction.* Endoscopy, 2016. **48**(2): p. 156-163.
6. Sato, T., K. Hara, N. Mizuno, S. Hijioka, H. Imaoka, T. Yogi, H. Tsutsumi, T. Fujiyoshi, Y. Niwa, M. Tajika, et al., *Type of combined endoscopic biliary and gastroduodenal stenting is significant for biliary route maintenance.* Intern. Med., 2016. **55**(16): p. 2153-2161.
7. Hamada, T., Y. Nakai, J.Y. Lau, J.H. Moon, T. Hayashi, I. Yasuda, B. Hu, D.-W. Seo, H. Kawakami, M. Kuwatani, et al., *International study of endoscopic management of distal malignant biliary obstruction combined with duodenal obstruction.* Scand. J. Gastroenterol., 2018. **53**(1): p. 46-55.
8. Debourdeau, A., F. Caillol, C. Zemmour, J.P. Winkler, C. Decoster, C. Pesenti, J.-P. Ratone, J.M. Boher, and M. Giovannini, *Endoscopic management of concomitant biliary and duodenal malignant obstruction: Impact of the timing of drainage for one vs. two procedures and the modalities of biliary drainage.* Endoscopic Ultrasound, 2021. **10**(2): p. 124-133.
9. Mangiavillano, B., R. Kunda, C. Robles-Medranda, R. Oleas, A. Anderloni, A. Sportes, C. Fabbri, C. Binda, F. Auriemma, L.H. Eusebi, et al., *Lumen-apposing metal stent through the meshes of duodenal metal stents for palliation of malignant jaundice.* Endosc Int Open, 2021. **9**(3): p. E324-E330.
10. Canakis, A., K.E. Hathorn, S.S. Irani, and T.H. Baron, *Single session endoscopic ultrasound-guided double bypass (hepaticogastrostomy and gastrojejunostomy) for concomitant duodenal and biliary obstruction: A case series.* J Hepatobiliary Pancreat Sci, 2022. **29**(8): p. 941-949.
11. Sasaki, T., T. Takeda, Y. Yamada, T. Okamoto, C. Mori, T. Mie, A. Kasuga, M. Matsuyama, M. Ozaka, and N. Sasahira, *Long-term outcomes of endoscopic double stenting using an anti-reflux metal stent for combined malignant biliary and duodenal obstruction.* J. Hepato-Biliary-Pancreatic Sci., 2023. **30**(1): p. 144-152.
